# Biomechanical Comparison of Three Different Fixation Methods for Unstable Basicervical Intertrochanteric Fractures Using a Novel Cephalomedullary Nail

**DOI:** 10.3390/medicina62020322

**Published:** 2026-02-04

**Authors:** Kyung-Jae Lee, Kyu Tae Hwang, Incheol Kook, Se-Won Lee, Sung-Jae Lee, Jin-Ho Yoon, Je-Hyun Yoo

**Affiliations:** 1 Department of Orthopedic Surgery, Keimyung University Dongsan Hospital, 1035 Dalgubeol-daero, Dalseo-gu, Daegu 42601, Republic of Korea; oslee@dsmc.or.kr; 2 Department of Orthopaedic Surgery, Hanyang University Seoul Hospital, 222 Wangsimni-ro, Seongdong-gu, Seoul 04763, Republic of Korea; hwangkt@hanyang.ac.kr (K.T.H.); nathan0319@naver.com (I.K.); 3 Department of Orthopedic Surgery, Yeouido St. Mary’s Hospital, College of Medicine, The Catholic University of Korea, Seoul 07345, Republic of Korea; ssewon@naver.com; 4 Department of Biomedical Engineering, Inje University, Gimhae 50834, Republic of Korea; 5 Department of Orthopaedic Surgery, Hallym University Sacred Heart Hospital, Hallym University College of Medicine, Anyang 14068, Republic of Korea

**Keywords:** femur, femoral neck fractures, hip fractures, fracture fixation, intramedullary, biomechanical phenomena

## Abstract

*Background and Objectives:* This biomechanical study aimed to compare the fixation stability of proximal fragments and assess the mechanical properties in models of unstable basicervical intertrochanteric fractures. *Materials and Methods:* Thirty-six synthetic femur models were utilized. After cephalomedullary nail insertion, unstable basicervical intertrochanteric fractures were created using an engraving machine. Specimens were divided into three groups based on the femoral head fixation method: Group 1 (n = 12, single 100 mm lag screw); Group 2 (n = 12, lag screw + 75 mm anti-rotation screw); and Group 3 (n = 12, lag screw + 95 mm anti-rotation screw). The anti-rotation screws were full-threaded locking screws positioned just below the lag screw. After applying 10,000 vertical cyclic loads, stereophotogrammetry was used to evaluate the proximal fragment rotation in three planes (coronal, sagittal, and axial), and screw-tip displacement was measured radiographically. Vertical load was then applied at a 10 mm/min rate until structural failure. *Results*: Rotational change in the sagittal plane was least in Group 3 (Group 1 = 1.7 ± 1.3°, Group 2 = 1.0 ± 0.8°, Group 3 = 0.6 ± 0.6°, *p* = 0.038). Varus (coronal plane) and retroversion (axial plane) collapse did not differ significantly among the three groups. While cranial migration showed no difference, axial migration was the significantly lowest in Group 3 (Group 1 = 1.07 ± 0.62 mm, Group 2 = 0.60 ± 0.57 mm, Group 3 = 0.50 ± 0.43 mm, *p* = 0.040). Failure load was slightly higher in Groups 2 and 3 than in Group 1, but without statistical significance. No significant differences were observed between Group 2 and Group 3 in any biomechanical outcomes. *Conclusions*: The novel cephalomedullary nail with a long inferior anti-rotation screw significantly reduced rotational instability and axial migration compared to a single-lag screw. There was no significant difference in the rotational stability between the 75 mm and 95 mm anti-rotation screw groups. This novel nail demonstrates superior biomechanical properties in this experimental model and warrants clinical evaluation for treating unstable basicervical intertrochanteric fractures.

## 1. Introduction

A basicervical intertrochanteric (IT) fracture is defined as a fracture in which the fracture line passes through the base of the femoral neck to its junction with the intertrochanteric region [1,2,3]. Basicervical IT fractures are biomechanically extracapsular fractures, but are considered more unstable than conventional IT fractures because of the lack of muscular attachment to the proximal fragment, the proximity of the fracture line to the femoral neck, and the paucity of the cancellous portion at the fracture site [1,2,4,5,6]. Cephalomedullary (CM) nails are recommended for treating basicervical IT fractures; however, there is a risk of fixation failure such as cut-out or cut-through after nailing due to high rotational instability of the proximal fragment [7,8,9,10].

To increase the rotational stability of the proximal fragment, an additional anti-rotation screw has been used along with a lag screw [5]. However, anti-rotation screws have been reported to cause a complication called the Z-effect or reverse Z-effect, in which the imbalance of the compressive force between the lag screw and the anti-rotation screw causes the proximal or distal migration of anti-rotation screw [11,12,13]. To date, most anti-rotation screws are partially threaded and inserted through the superior femoral neck region above the lag screw. The use of these anti-rotation screws can result in insufficient resistance against the rotational instability of the proximal fragment because these relatively thin screws are inserted into the superior femoral neck, which has poor bone quality [14].

**Figure 1:**
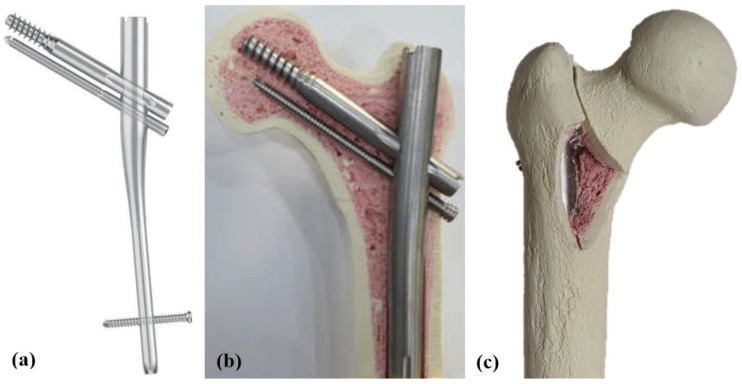
The novel cephalomedullary (CM) nail and the artificial femur model used in this study. (**a**) Schematic illustration of the Osteonoic Femoral Anti-rotation Nail (OSFAN); the anti-rotation screw is located below the lag screw, and it is a fully threaded screw fixed to the nail body with a locking mechanism. (**b**) Coronal section of the OSFAN when inserted into an artificial femur model. Note the insertion of a full-length (95 mm) inferior anti-rotation screw. (**c**) Posterior view of the specimen with the OSFAN inserted and basicervical intertrochanteric fracture (ITF) created corresponding to AO/OTA 31-A2.2; note the removal of the posteromedial fragment including the lesser trochanter. Full-length (95 mm) inferior anti-rotation screw was inserted.

Therefore, we developed a novel nail design for treating basicervical IT fractures to decrease the rotational instability of the proximal fragment and the high risk of fixation failure. This novel nail, called Osteonic Femoral Anti-rotation Nail (OSFAN^®^; Osteonic Inc., Seoul, South Korea), provides increased rotational stability in the proximal fragment by inserting an anti-rotation screw 5 mm below the lag screw, which is placed at the inferior femoral neck with relatively better bone quality [14]. This anti-rotation screw was designed to be fully threaded and locked with the nail body via a thread at screw hole to avoid the imbalance of compressive force between two screws and reduce its migration (Figure 1). Subsequently, this screw can minimize the collapse of the proximal fragment and maintain the original femoral offset as much as possible after initial compression at the fracture site.

We sought to determine whether the addition of inferior anti-rotation screws improve rotational stability and mechanical properties compared to single-lag screws. We found that, in cases of narrow femoral neck, when placing an anti-rotation screw after a lag screw, it may not pass through the femoral neck region. The width of the femoral neck varies by race and gender, with differences of up to 16% [15]. Therefore, it is necessary to determine whether there is a difference in biomechanical stability when not using an anti-rotation screw and when using a short one, under the assumption that this screw cannot pass through the femoral neck region when using the novel nail in patients with a narrow femoral neck.

This biomechanical study aimed to compare the fixation stability of the proximal fragments and their mechanical characteristics in models of unstable basicervical IT fracture, which were stabilized using a newly developed CM nail with three different types of femoral head fixation. We hypothesized that a full-length inferior anti-rotation screw would provide superior rotational stability compared to a lag screw alone or a short anti-rotation screw.

## 2. Materials and Methods

### 2.1. Specimen Preparation

For this biomechanical study, 36 composite femurs with a customized density were used (Sawbones; Pacific Research Laboratories, Vashon, WA, USA). Each synthetic femur was overlaid with a thin cortical layer and filled with low-density polyurethane foam to simulate osteoporotic human bone. The models had a length of 470 mm, a neck-shaft angle of 125°, an anteversion of 15°, a head diameter of 48 mm, and a canal diameter of 16 mm [16]. This biomechanical study did not require institutional review board approval because it did not involve human or cadaveric subjects.

The novel nail employed in this study was made from a titanium alloy measuring 200 mm in length with a proximal diameter of 15.5 mm. The lag screw was partially threaded with a diameter of 10.5 mm and featured a negative flank profile angle to enhance fixation by converting shear force into compressive force. An anti-rotation screw, inserted just below the lag screw within the femoral head, was fully threaded with three distinct diameters: 5.5 mm at the femoral head and neck fixation part, 6.3 mm at the nail assembly part, and 7.0 mm at the lateral cortex. This screw’s thread engaged with the corresponding thread in the nail’s screw hole and was secured with a 6.0 Nm torque-limiting driver at the lateral cortex upon final insertion (Figure 1).

All implants were inserted using a standard technique under fluoroscopic guidance with a specialized jig and a targeting device [16]. Under C-arm guidance, the entry point was positioned at the tip of the greater trochanter (GT) on the anteroposterior (AP) view and at the midline of the proximal femur on the lateral view. After proximal reaming along a guide pin, the nail was assembled to the targeting device and inserted. The lag screw was then positioned at a center-to-center location approximately 10 mm below the apex of the femoral head in both the anteroposterior and lateral views under fluoroscopy. A lag screw length of 100 mm was selected for all femur models. All specimens were stabilized with a CM nail of uniform centrum-collum-diaphyseal angle (125°) and diameter (12 mm), with the lag screws and distal locking screws being of consistent length across all models. The proximal reaming diameter of the nail was 15.5 mm, and 13 mm diameter was performed for the nail body. No torque-limiting driver was used during the insertion of the nail body or lag screw. C-arm images were archived to document the nail’s position during implantation. Implantation was performed by three orthopedic surgeons with over 10 years of experience using IM nails. Surgeons used the same jig and targeting device, and consensus was established by comparing the archived C-arm images after implantation.

**Figure 2:**
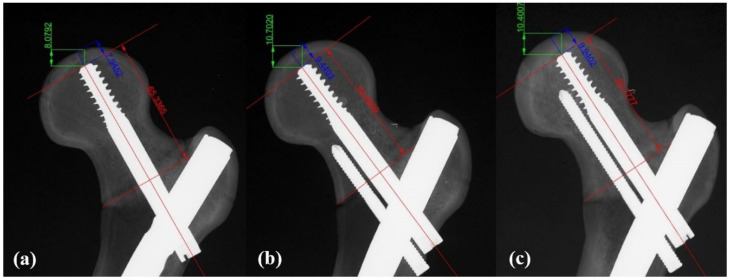
Anteroposterior radiographs showing the novel cephalomedullary (CM) nailing with three different fixation methods: (**a**) lag screw (100 mm) only, (**b**) lag screw with a short inferior anti-rotation screw (75 mm), and (**c**) lag screw with a long inferior anti-rotation screw (95 mm); the short anti-rotation screw does not pass through the femoral neck region and ends at the narrowest part of the femoral neck. Sliding distance between red arrows, cranial migration between green arrows, axial migration between blue arrows.

The tip-apex distance (TAD) was confirmed to be less than 25 mm in both AP and axial views for all specimens. TAD measurements were performed by two orthopedic surgeons who did not participate in the implantation. Discrepancies were resolved through discussion. The TAD values ranged from 18 to 22 mm, with Group 1 having a TAD of 20.2 ± 0.6 mm, Group 2 having a TAD of 19.9 ± 0.4 mm, and Group 3 having a TAD of 20.3 ± 0.2 mm. The 36 femur models were divided into three groups for femoral head fixation: Group 1 with a lag screw alone (n = 12), Group 2 with a lag screw and a 75 mm anti-rotation screw (n = 12), and Group 3 with a lag screw and a 95 mm anti-rotation screw (n = 12) (Figure 2). For distal fixation, an interlocking screw with a diameter of 5.0 mm and a length of 40 mm was used in all specimens.

After nail insertion in all femur models, unstable basicervical IT fractures corresponding to AO/OTA type 31-A2.2 were homogenously created using an engraving machine (Shin-il Inc., Busan, Korea) based on pre-designed specifications [16,17]. The fracture gap was set at 2 mm, with the main fracture line originating at the base of the femoral neck, extending to its junction into the intertrochanteric region. Additionally, a posteromedial fragment measuring 6 × 4 cm, which included the lesser trochanter, was removed from all femoral models (Figure 1).

### 2.2. Biomechanical Testing

**Figure 3:**
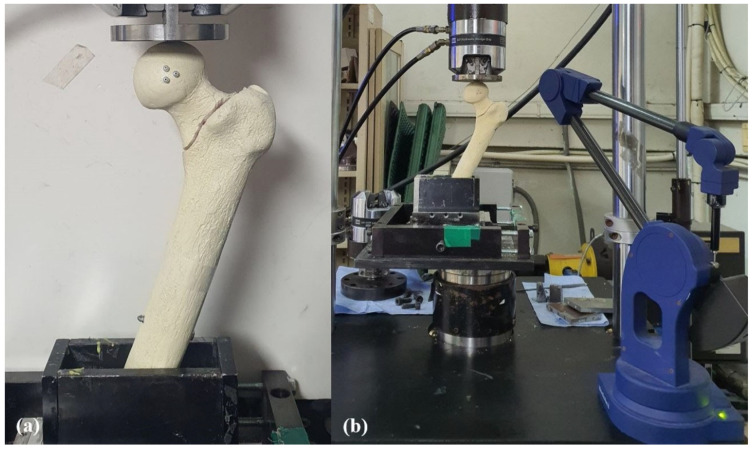
Setup of the biomechanical test. (**a**) The specimen was fixed adduction of 25° in the coronal plane, and three plastic beads were attached to the anterior surface of the femoral head in a triangular pattern. (**b**) Before and after the axial cyclic loading test, three-dimensional coordinates of the beads were measured using a MicroScribe M digitizing system (Revware Inc., Raleigh, NC, USA) positioned at 30° to the center of the specimen, from which the rotation of the proximal fragment in each plane was calculated.

In preparation for biomechanical testing, the specimen was removed 30 cm distal to the tip of the GT using a custom three-dimensional (3D)-printed cutting frame. This step was necessary to prevent potential femoral shaft fractures during loading tests. The femur models were then secured on a steel square holder with resin to ensure stability during testing [18]. Each specimen was positioned at neutral in the sagittal plane and 25° adduction in the coronal plane to simulate a single-leg stance [19] (Figure 3). A servo-hydraulic test machine (MTS 858 Mini Bionics, MTS Systems, Eden Prairie, MN, USA) was used for this biomechanical study. A flat polished applicator was utilized to allow for the femoral head to move under load [20]. Prior to the cyclic load test, three black markers, each 1 mm in diameter, were attached in a triangular pattern to the anterior surface of the femoral head to facilitate the tracking of movement (Figure 3).

Preliminary tests were conducted to identify the appropriate load for cyclic vertical loading on the native femoral bone models. The preliminary tests were conducted three times, starting at 1400 N and progressively decreasing the load to 1100 N and then 750 N. Initially, a load of 1400 N was tested, but due to femoral shaft fractures occurring at loads between 1100 and 1200 N, the final load was reduced to 750 N referencing prior biomechanical studies [21,22]. Although the maximum cyclic load of 750 N is lower than physiological peak loads, it was selected to prevent the premature failure of the synthetic bone model while ensuring a reliable relative comparison between the groups. The loading procedure commenced with a 100 N preload, applied at a rate of 20 N/min, to ensure proper contact between the femoral head and the testing apparatus [4,16]. Afterward, the specimens underwent cyclic vertical loading ranging from 75 to 750 N at a rate of 2 Hz for 10,000 cycles, simulating walking conditions approximately six weeks post-surgery [21,23]. Finally, a continuous vertical load was applied at a rate of 10 mm/min until the construct experienced failure, and the load–displacement curve was recorded throughout this process [21]. Failure was defined by several parameters, such as femoral neck fracture, implant failure, cut-out, fragment displacement exceeding 15 mm, or a sudden drop in load resistance on the load–displacement curve [18]. The displacement was measured from the femoral head apex.

**Figure 4:**
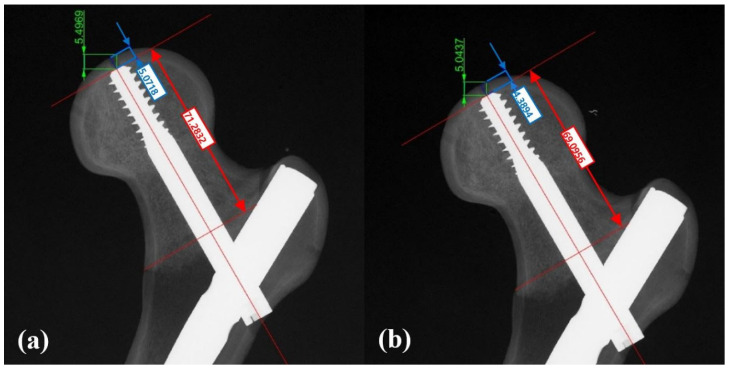
Anteroposterior plain radiographs of the specimen (**a**) before and (**b**) after axial cyclic loading were taken to determine the migration of the lag screw tip within the femoral head in the screw axis (axial, line between blue arrows) and cranial directions (line between green arrows) using a computer-assisted calibration program (Rhinoceros 3, Robert McNeil & Associates, Seattle, WA, USA), Sliding distance between red arrows.

After cyclic loading and a five-minute relaxation period, anteroposterior radiographs were taken to assess lag screw migration. Radiographs were calibrated using a 155 mm reference plastic rod. Migration in the axial and cranial directions was measured using a computer-aided design program (Rhinoceros 3, Robert McNeel & Associates, Seattle, WA, USA) (Figure 4) [18].

**Figure 5:**
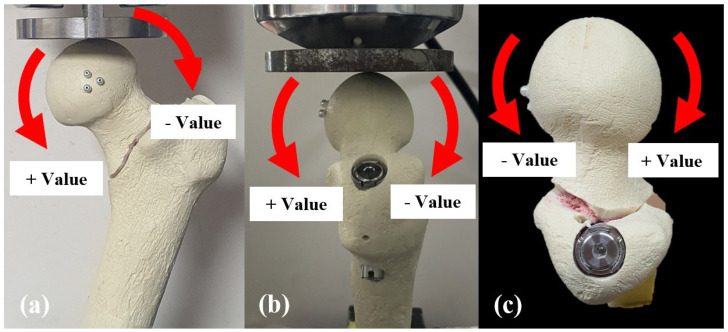
Rotation measurements in each plane. (**a**) Varus change in the coronal plane was defined as positive. (**b**) Counter-clockwise rotation in the sagittal plane as seen from the lateral side of the specimen was defined as positive. (**c**) Retroversion change in the axial plane was defined as positive.

Rotation of the proximal fragment after the cyclic loading test was assessed in the coronal, sagittal, and axial planes based on the position of three markers on the femoral head using the Microscribe M digitizing system (Revware Inc., Raleigh, NC, USA) and Bryant angles [24,25]. The Microscribe M digitizing system was connected to a computerized system for measurement and calculation. The Microscribe system was placed 30° from the center of the specimen to measure the 3D coordinates of the markers before and after cyclic loading (Figure 3), with the system’s accuracy reported as ± 0.05 mm [26]. In the coronal plane, rotation was categorized as either varus or valgus, with varus rotation defined as varus collapse. In the sagittal plane, viewed from the lateral side of the femur, rotation was classified as counter-clockwise or clockwise, with counter-clockwise rotation defined as sagittal rotation. In the axial plane, rotation was categorized as retroversion or anteversion, with retroversion defined as retroversion collapse. For analysis, rotation in the direction corresponding to varus, counter-clockwise, and retroversion was recorded as a positive value in each respective plane (Figure 5).

### 2.3. Statistical Analysis

Comparative analysis was performed among the three groups. First, a test for normality was performed using the Shapiro–Wilk test. When the normality was satisfied, one-way analysis of variance and a Tukey–Kramer post hoc test were used. The Kruskal–Wallis test and a Mann–Whitney post hoc test were used to evaluate the differences among the three groups for the variables studied. All statistical analyses were conducted using SPSS version 27 (SPSS Inc., Chicago, IL, USA) with mean and standard deviation results. A two-tailed *p*-value of less than 0.05 was considered statistically significant, and for post hoc tests, the significance level was adjusted to *p* < 0.017 (0.05/3) using Bonferroni’s correction. An a priori power analysis, based on previous studies from both the literature and our own laboratory, indicated that a sample size of 8 per group would provide 80% power at a significance level of 0.05.

## 3. Results

For the mean failure load, Group 1 was 2727 ± 565 N (95% confidence interval (CI) 2368—3086 N), Group 2 was 2861 ± 539 N (95% CI 2518—3203 N), and Group 3 was 3195 ± 430 N (95% CI 2745—3109 N). Group 2 and 3 showed 4.9% and 17.1% higher failure loads, respectively, than Group 1, but there was no significant difference among the three groups (*p* = 0.087) (Table 1). Post hoc analysis showed no significant differences between Groups 1 and 2, between Groups 2 and 3, and between Groups 1 and 3 (*p* = 0.333, 0.120 and 0.032, respectively, applying Bonferroni’s correction). Regarding the failure mode, excessive displacement of the proximal fragments by more than 15 mm was observed in 33 of the 36 synthetic bone-implant constructs. In the remaining three specimens, the proximal fragment experienced fractures, with two cases occurring in Group 1 and one in Group 2.

Cranial migration of the lag screw after cyclic loading was less in Groups 2 (0.79 ± 0.71 mm) and 3 (0.57 ± 0.36 mm) than Group 1 (0.98 ± 0.59 mm) by 15% and 42%, respectively. However, there was no significant difference among the three groups (*p* = 0.264). Meanwhile, axial migration of the lag screw showed a significant difference among the three Groups (*p* = 0.040). Group 3 exhibited a 53% lower value compared to Group 1, with the post hoc test revealing a significant difference between these two groups (*p* = 0.014, applying Bonferroni’s correction). However, no significant differences were found between Groups 1 and 2 or between Groups 2 and 3 (*p* = 0.060 and 0.799, respectively, applying Bonferroni’s correction). One more important finding to note is that there was no cut-out of the lag screw during the cyclic loading test and no Z- or reverse Z-effect was observed.

The rotation of the proximal fragment was measured along three planes. There was a significant difference in sagittal plane rotation between the three Groups (*p* = 0.038), and the post hoc tests showed that Group 3 was significantly smaller than Group 1 (*p* = 0.010, applying Bonferroni’s correction). There were no significant differences between Groups 1 and 2 or between Groups 2 and 3 (*p* = 0.128 and 0.347, respectively, applying Bonferroni’s correction). Additionally, no significant differences were observed among the three groups for varus collapse along the coronal plane (Group 1 = 1.8 ± 1.1°, Group 2 = 1.4 ± 1.7°, Group 3 = 1.3 ± 1.1°, *p* = 0.419) or retroversion collapse along the axial plane of the proximal fragment (Group 1 = 0.5 ± 0.5°, Group 2 = 0.5 ± 0.4°, Group 3 = 0.4 ± 0.3°, *p* = 0.916) (Table 1).

## 4. Discussion

In this biomechanical study, we found that: (1) A long (95 mm) inferior anti-rotation screw significantly reduced sagittal rotation by 65% compared to the lag screw alone. (2) Axial migration was significantly less with the long anti-rotation screw. (3) No Z-effect was observed. These findings suggest that the group using 95 mm full-length anti-rotation screw demonstrated the highest construct stability and failure load among the three groups. In a biomechanical study by Kwak et al., which compared the stability of three types of CM nails based on proximal fixation methods (lag screw, hybrid lag screw with blade, and blade type) in a simulated unstable basicervical IT fracture model, the lag-screw-type CM nail exhibited significantly lower rotational stability, particularly in the sagittal plane [16]. In our study, the average sagittal rotation angle in the lag-screw-alone group was 1.7 degrees, whereas the addition of a 95 mm anti-rotation screw significantly improved the sagittal plane stability by reducing the sagittal rotation angle by 65%. This indicates that the use of a full-length anti-rotation screw in our new instrumentation is the most efficient way to enhance rotational stability. However, there was no significant difference in the rotational stability between the 75 mm and 95 mm anti-rotation screw groups. Although adding the full-length anti-rotation screw to the OSFAN^®^ implant is recommended in unstable basicervical IT fractures, the addition of a shorter anti-rotation screw may also enhance the rotational stability of the proximal fragment, especially in patients with a narrow femoral neck where inserting a full-length screw is not feasible.

Although the incidence of a basicervical IT fracture is rare [2], these fractures are associated with a high rate of fixation failure such as cut-out or cut-through [8]. This may be due to the inherent instability of the proximal fragment, characterized by the narrow cortical base of the proximal fragment and subsequent contact area, with insufficient cancellous interdigitation at the main fracture site compared to conventional IT fracture [8,27]. While CM nails are commonly used to treat unstable basicervical IT fractures, the clinical outcomes are generally inferior to those for conventional IT fractures due to the fracture pattern itself [2,8]. Watson et al. reported a fracture union rate of 45%, an implant failure rate of 45%, and a nonunion rate of 5%, despite all cases achieving an appropriate TAD of ≤ 25 mm and anatomical reduction [8]. These findings suggest that factors other than surgical techniques may contribute to the high failure rates in unstable basicervical IT fractures. Therefore, to ensure adequate biomechanical stability, the optimal implant for these complex fractures must be able to withstand weight-bearing load and maintain the rotational stability for relatively short proximal fragments. Accordingly, the fixation type of the femoral head in these fractures should provide greater purchasing power to resist the rotational instability of the proximal fragment. Massoud reported that the use of a single-lag screw for CM nail fixation in basicervical IT fractures was insufficient to control the rotational instability of the proximal fragment, and the additional anti-rotation screw showed greater rotational stability [5]. Other studies also reported that the rotational stability of the proximal fragment is an important consideration for implant selection in unstable basicervical IT fractures [7,28].

For these reasons, we designed a new implant for unstable basicervical IT fractures, which has a cephalomedullary fixation method incorporating a central lag screw and an inferior full-threaded anti-rotation screw within the femoral head. To prevent the Z-effect, which has been reported as a complication of existing IM nails using an anti-rotation screw, we inserted a full-threaded anti-rotation screw that does not provide compressive force to avoid an imbalance in the compressive force between the lag screw and anti-rotation screw [11,12]. Additionally, an anti-rotation screw was inserted into the inferior femoral neck and calcar femorale regions, which have better bone quality, to improve fixation and a locking mechanism via the thread at the connection between the CM nail body and the anti-rotation screw could be applied to minimize the collapse of the proximal fragment and subsequently maintain the femoral offset as much as possible [11,12,14]. Future biomechanical studies comparing the inferior anti-rotation screw with the conventional superior anti-rotation screw are needed to more specifically confirm the function of the inferior anti-rotation screw.

The axial migration was also significantly least in the lag screw combined with the full-length anti-rotation screw group among three groups, although there was no significant difference in the cranial migration of the lag screw. The 95 mm anti-rotation screw group showed 53% and 20% less axial migration of lag screw than the lag-screw-only group and 75 mm anti-rotation screw group, respectively. The rotation of the proximal fragment in IT fractures generally causes this migration of the lag screw within the femoral head. Subsequently, the greater rotational instability of the proximal fragment results in the greater migration of the lag screw. We believe that these results are attributed to the enhanced rotational stability of the proximal fragment caused by the insertion of the full-length anti-rotation screw and the additional resistance to axial forces on the screw provided by securing the anti-rotation screw to the nail body with a locking mechanism. In previous studies, locked screws showed higher torsional stability and failure strength than non-locked screws [29,30]. It has also been reported that locked screw fixation provides a more equally distributed load over the entire construct [31]. Taken together, the decreased pull-out of the anti-rotation screw and 53% less axial migration compared to the lag screw alone in this biomechanical study suggest that the locking fixation of the anti-rotation screw in our novel implant contributed to the highest rotational stability of the proximal fragment. In addition, in the current study, no mechanical failures, such as bending or breakage or the Z-effect of the anti-rotation screw, were observed. We believe that these results are due to the insertion of an anti-rotation screw inferior to the thicker lag screw and the locking fixation of the anti-rotation screw with both the nail body and lateral cortex.

When treating hip fractures with CM nail, closed reduction is performed in most cases, and anatomical reduction can be achieved. However, in highly unstable fractures such as basicervical IT fractures, closed reduction may fail, necessitating open reduction. It has been reported that the risk of requiring open reduction is significantly increased in unstable fractures such as AO classification 31-A2.3, A3.2, A3.3, when the lateral wall is disrupted, when there is a posterior wall fracture, when the proximal fragment is flexed, or when the calcar femorale is disrupted [32].

The limitations of this study include the use of an artificial femur model, which may produce different outcomes from actual in vivo biomechanical results. The artificial femur model used in this study had a uniform density throughout the model. The human femur has different bone qualities depending on the region, and, in most cases, the inferior region of the femoral neck shows better bone quality than the superior region. This density difference between the artificial femur model and a human femur may cause an underestimation of the effect of the inferiorly inserted anti-rotation screw in this biomechanical study. Furthermore, the use of synthetic bone has the disadvantage of not simulating the actual bone union process. In this study, the fracture line was created after IM nail implantation, forming a consistent 2 mm fracture gap. In reality, the fracture is reduced before inserting the IM nail, and the fracture site is often compressed after lag screw insertion. Although this study’s approach differs somewhat from clinical practice, we created a fracture gap of a constant width after IM nail implantation to maintain consistency in all other conditions except the inferior anti-rotation screw, thereby enhancing data reliability. This methodology limits the transferability of the study results to clinical settings. This study is a biomechanical investigation without clinical data, thus requiring additional clinical validation. This study’s protocol also has limitations. Only a single loading rate (10 mm/min) was applied in the failure load test. Scenarios such as sudden lateral impact occurring during a fall were not simulated. The cyclic load test was performed only up to 10,000 cycles; fatigue testing beyond this was not conducted. The 10,000 cycles simulated 6 weeks post-surgery, but the healing process often takes longer, and the situation beyond 6 weeks cannot be known precisely. In addition, this study applied only axial loading and examined the results by simulating a single-leg stance. Other physiological positions, such as walking, climbing stairs, or sitting down and standing up, were not tested. Forces in different directions are generated by the surrounding muscles during gait and are much more complex than the loading condition in this study. Additionally, the measurement error of the measuring system may have slightly affected the results, including the rotation angle.

However, the strengths of this study include a sample size of 12 specimens per group, which ensured sufficient statistical power. In addition, the use of identical synthetic femur models and the creation of consistent fracture patterns have enhanced the reliability of the data. The accuracy of the results was further improved by employing a precise 3D digitizing system with an error margin of only 0.05 mm. Therefore, we believe that the current study suggests that this newly designed CM nail is a viable option for enhancing fixation stability and reducing fixation failure in the treatment of unstable basicervical IT fractures.

## 5. Conclusions

In this biomechanical study of the use of OSFAN^®^ for unstable basicervical IT fractures, the combination of lag screws and long anti-rotation screws significantly increased the rotational stability of the implant and decreased axial migration compared to cephalomedullary fixation with lag screws alone. Additional biomechanical studies and large-scale prospective multi-center studies are required to confirm our results and verify the clinical utility of the newly designed CM nail for unstable basicervical IT fractures. This novel nail with a lag screw and a long inferior anti-rotation screw demonstrates superior biomechanical properties in this experimental model and warrants clinical evaluation for treating unstable basicervical IT fractures.

## Figures and Tables

**Table 1:** Results of the biomechanical tests with three fixation methods.

Variables	Group 1 (n = 12)	Group 2 (n = 12)	Group 3 (n = 12)	*p*-Value
Failure load (N)	2727 ± 565	2861 ± 539	3195 ± 430	0.087 *
Cranial migration (mm)	0.98 ± 0.59	0.79 ± 0.71	0.57 ± 0.36	0.264 ^†^
Axial migration (mm)	1.07 ± 0.62	0.60 ± 0.57	0.50 ± 0.43	0.040 ^†^
Rotation (°)				
Coronal Plane (Varus: +)	1.8 ± 1.1	1.4 ± 1.7	1.3 ± 1.1	0.419 ^†^
Sagittal Plane (Counter-clockwise: +)	1.7 ±1.3	1.0 ± 0.8	0.6 ± 0.6	0.038 ^†^
Axial Plane (Anteversion: +)	0.5 ± 0.5	0.5 ± 0.4	0.4 ± 0.3	0.916 ^†^

Data are presented as mean ± standard deviation. * One-way analysis of variance. ^†^ Kruskal–Wallis test.

## Data Availability

The original contributions presented in this study are included in the article. Further inquiries can be directed to the corresponding author.
